# Health Care Utilization Among Patients With Coronary Artery Disease Using mHealth Wearable Devices: Feasibility Observational Study

**DOI:** 10.2196/81922

**Published:** 2026-11-13

**Authors:** Indranil Bardhan, Gari Clifford, Mujeeb Basit, DuWayne Willett, Tianjian Guo, Herman Taylor

**Affiliations:** 1 McCombs School of Business, The University of Texas at Austin, 2110 Speedway, B6500, Austin, TX, 78705, United States, 1 512-232-2501; 2 Department of Biomedical Informatics, Emory University, Atlanta, GA, United States; 3 Cardiology, Internal Medicine, University of Texas Southwestern Medical Center, Dallas, TX, United States; 4 Cardiology, University of Texas Southwestern Medical Center, Dallas, TX, United States; 5 Kelley School of Business, Indiana University, Bloomington, IN, United States; 6 Cardiovascular Research Institute, Morehouse College, Atlanta, GA, United States

**Keywords:** wearable devices, mHealth, patient engagement, physiological data, health care utilization

## Abstract

**Background:**

Although prior research has explored patient engagement with health care providers through software applications such as patient portals, the impact of patient engagement with mobile health (mHealth) technologies on subsequent utilization of health care resources remains poorly understood. Specifically, it remains unclear whether physiological data collected from wearable devices can be linked to future health care encounters across different modes of care utilization.

**Objective:**

This pilot study was aimed at evaluating the feasibility of an mHealth prototype for analyzing cardiovascular disease outcomes and health care utilization among patients using digital health interventions. Specifically, we examined the feasibility of integrating physiological data from wearable devices to examine associations with future care utilization, offering early evidence for the effectiveness of patient engagement with mHealth platforms.

**Methods:**

Patients with a history of coronary artery disease were recruited from the cardiology clinic of a large academic medical center. Before data collection, the study coordinator trained 20 patients on the use of Garmin devices, of whom 19 were included in the analyses. Data collected from the use of wearable devices were analyzed together with electronic health record data. Patient health outcomes were tracked from June 2019 to May 2021, including approximately 1500 health care encounters that encompassed hospital and outpatient visits, telehealth visits, and other encounters. Beyond establishing the feasibility of the mHealth platform, this work provided preliminary evidence of associations between (1) mHealth device use and future health care utilization and (2) physiological data and the frequency of future health care encounters.

**Results:**

This study demonstrated the feasibility of an mHealth prototype for collecting and analyzing data to measure care utilization among patients with cardiovascular disease. Participants’ use of the wearable mHealth device was associated with a statistically significant lower frequency of subsequent hospital and outpatient visits but greater utilization of other health care services, such as procedures, infusions, and medication refills, during the study period. However, none of these associations were statistically significant when the pretreatment period was restricted to the COVID-19 era. Minimum heart rate was associated with a higher frequency of future hospital and outpatient utilization, whereas activity time was associated with a higher frequency of procedural and refill visits.

**Conclusions:**

This pilot study provides preliminary evidence that mHealth device use is associated with a reduction in future inpatient and outpatient visits and an increase in the number of visits for other health care services. It also indicates that patients’ physiological data, such as minimum heart rate and activity time, are associated with the frequency of future health care encounters. These findings should be interpreted as exploratory signals rather than statistically definitive conclusions. This study provides proof of concept that an integrated mHealth platform can generate physiologically meaningful measures associated with subsequent care utilization, supporting its potential as a scalable tool for patient engagement and care coordination.

## Introduction

This research was aimed at evaluating the feasibility of a novel mobile health (mHealth) technology prototype for studying cardiovascular disease by supporting patients in embracing behaviors that may improve cardiovascular health through innovative digital health strategies. We developed a novel, scalable approach to monitor and improve health behaviors among patients with cardiovascular disease using a smartphone app. This approach was designed to be unobtrusive and require little to no user input, except when participants chose to engage with the app.

The overall goal of this research was to improve patient engagement and care coordination with health care providers by using mHealth devices for chronic disease management. Specifically, our objectives were to (1) establish the feasibility of an mHealth prototype using “in the wild” data sourced from participants’ use of wearable devices and (2) evaluate the relationships between patient engagement, use of mHealth devices, and future health care utilization.

Previous studies on patient engagement and care coordination have typically explored the use of tethered patient portals to assess patient engagement with care providers and its association with future health outcomes and utilization of health care resources [1]. Recent advancements in mobile technologies and cloud computing suggest that the use of smartphone apps is associated with a statistically significant increase in the 6-minute walk test distance [2,3]. Specifically, an observational study, based on a large cohort of over 30,000 users, reported significant associations between cardiovascular disease status (including peripheral artery disease, atrial fibrillation, and pulmonary arterial hypertension) and the 6-minute walking distance [4]. A related study showed that actigraphy data passively collected from smartphones can help differentiate decompensation events from euvolemic states among veterans with heart failure, with minimal cost and no user input [5]. Further, a randomized controlled trial involving 1070 patients with diabetes in Asia showed that adoption of an mHealth app was associated with improvements in both short-term metrics (reductions in blood glucose and glycated hemoglobin levels) and longer-term metrics, such as hospital visits and medical expenses [6].

However, a notable gap remains in our understanding of how patients with coronary artery disease (CAD) use smartphone-based health apps to monitor their lifestyles and engage with care providers [7]. Specifically, the association between patients’ use of app-based wearable devices and patterns of future health care encounters, including hospital, emergency department, and outpatient visits, remains unclear and has not been empirically investigated [8,9]. For example, how does the use of wearable devices impact their future utilization of health care services, including hospital and emergency department visits as well as outpatient visits? Prior research has also not provided conclusive evidence regarding the relationship between the use of mHealth devices or apps that track patients’ physiological data and the incidence of future health care encounters [10].

Our study aimed to address this research gap and demonstrate the feasibility of a novel mHealth prototype for patient engagement and care coordination in a cohort of 20 patients with CAD who were followed closely during the study period. Specifically, we examined the association between patients’ use of the prototype mHealth wearable device and their overall engagement with health care providers. The mHealth device records essential physiological data, including blood pressure, heart rate, stress levels, sleep metrics, and physical activity. This streaming data feed is then matched with patients’ electronic health record (EHR) data through a cloud-based platform for near real-time analytics. We assessed the viability of the mHealth prototype as a platform for patient-provider engagement by evaluating the relationships between physiological data collected through wearable devices and health care utilization in inpatient and outpatient settings. Beyond establishing the feasibility of the mHealth platform, we aimed to provide preliminary evidence of associations between mHealth device use and patient engagement and between specific types of physiological data and the frequency of future health care encounters. These analyses provide early insights into the potential association between mHealth interventions and health care utilization.

## Methods

### Methodology

We collected demographic, physiological, and clinical data from patients diagnosed with CAD. We then used statistical techniques to examine the associations between patients’ physiological data collected through their engagement with wearable devices and their future utilization of health care resources. Our statistical analyses followed a 2-step approach, as described below.

In the first step, we characterized patients’ patterns of health care engagement using EHR data. These data encompassed the timing and frequency of patients’ interactions with health care providers, including inpatient, outpatient, and telehealth encounters, as well as other visits such as for medication refills, procedures, and infusions. The initial study protocol also proposed collecting additional information, including measures derived from text messages, food choices, and monthly vital signs, as well as administering a survey at the end of the study. However, disruptions caused by the COVID-19 pandemic prevented these data from being collected as planned. These protocol deviations are described in the addendum to Multimedia Appendix 1.

In the second step, we collected patients’ physiological data, based on their use of mHealth wearable devices, and matched these data to their health care utilization records across the 4 types of encounters. We then fitted a generalized linear mixed model (GLMM, Equation 1) with a Poisson distribution and log link to conduct a prerecruitment-postrecruitment comparison aimed at evaluating whether patient enrollment in the mHealth platform was associated with changes in future health care utilization. The GLMM extends the standard generalized linear model framework by including patient-specific random intercepts along with fixed effects, accounting for within-patient correlation arising from repeated weekly observations over time. In this setting, the random intercept captures unobserved, time-invariant heterogeneity across patients, allowing the model to isolate the association between mHealth enrollment and health care utilization while accounting for patient-specific factors.


(1) $$\begin{array}{l} Visit_{it}∼Poisson\left(\mu_{it}\right),\log\left(\mu_{it}\right)=\\ \;\beta_{0}+\beta_{1}*Enroll_{it}+u_{i}u_{i}∼N(0,\sigma_{u}^{2}) \end{array}$$

Next, we conducted additional regression analyses to examine how patients’ physiological activity measures, collected from their use of mHealth devices, are associated with health care utilization. Specifically, we regressed the frequency of each of the 4 types of health care encounters on physiological measures derived from their use of wearable devices, while incorporating patient fixed effects to control for unobserved, time-invariant heterogeneity across patients. The empirical model is presented in Equation 2:


(2) $$\begin{array}{l} Encounters_{it}=f(Steps_{it},ActiveTime_{it},\\ MinHeartRate_{it},MaxStress_{it},Sleep_{it})+\;\alpha_{i} \end{array}$$

where the dependent variable represents the number of health care encounters of each type (ie, hospital visits, outpatient visits, telehealth visits, and other visits) for patient *i* in week *t*. All explanatory variables were measured as weekly averages. Specifically, *Steps* was measured in thousands of steps, *ActiveTime* in hours, *MinHeartRate* in beats per minute, *MaxStress* on the Garmin 1‐100 scale, and *Sleep* in hours. The term *α_i_* represents patient fixed effects, which account for time-invariant, unobserved patient-specific characteristics (such as demographic data) without these variables explicitly included in the model.

As the dependent variable is a count, we used a Poisson GLMM regression model with conditional fixed effects to estimate the model presented in Equation 2. In addition to estimating models for contemporaneous associations within the same week, we estimated the model in Equation 2 using temporal leads of 1 to 4 weeks. To account for potential false discoveries, we applied the Benjamini-Hochberg false discovery rate (FDR) correction jointly across the complete family of 100 tests: 4 health care utilization outcomes (hospital, outpatient, telehealth, and other visits) × 5 physiological measures (total steps, total active time, minimum heart rate, maximum stress, and total sleep time) × 5 temporal alignments (week *t* through week *t*+4).

In summary, the overall methodology involved three steps: (1) time trend analyses of hospital and outpatient utilization before and after patient enrollment, (2) comparisons of health care encounters before and after patient recruitment, and (3) regression analyses of patient health care outcomes based on panel data on patient characteristics and physiological measures collected from wearable sensors and EHRs. Given the small sample size, our analyses were exploratory and intended to (1) test the viability of the mHealth prototype and (2) evaluate associations between physiological data collected through prototype use and future health care utilization.

### Recruitment

We recruited patients with a history of CAD from the cardiology clinic of a large academic medical center in Texas. Initial screening for eligibility was based on data within the EHR system at the medical center. The eligibility criteria were as follows: (1) a record of at least one CAD-related visit in the cardiology clinic, (2) age greater than 21 years, (3) access to an Android smartphone, and (4) willingness to provide informed verbal consent based on the study protocol (Multimedia Appendix 1). Thus, eligible patients had a documented history of CAD based on their medical records. However, other information such as CAD severity and other clinical comorbidities, beyond baseline patient vitals, was not collected.

Next, the study research coordinator contacted patients who met the eligibility criteria and consented to participate. This resulted in a cohort of 20 patients, including 11 male and 9 female participants. All participants received a secure message from the study coordinator through the Epic MyChart app explaining the study requirements. First, the participants were required to use the Garmin vívosmart 4 wearable device, which was mailed to them at no cost, at all times during the study period. Second, they were asked to install 2 software apps on their Android smartphones based on guidance provided by the study coordinator. Third, they were required to participate in 2 video calls with the study coordinator to review the study objectives and requirements and receive assistance (if necessary) with installing the apps. Lastly, participants were asked to participate in several follow-up phone calls to answer any questions and verify that the smartphone apps were working properly. The Garmin vívosmart 4 was the primary mHealth device deployed to collect and process physiological data associated with participants’ activities. Participants were required to use the wearable device at all times during the study period to ensure that their physiological data could be continuously collected and monitored.

Once participant training was completed, data collection commenced immediately. As the study was aimed at examining the association between the use of wearable mHealth devices and patient engagement and care utilization, measures were adopted to protect the integrity, confidentiality, and privacy of patient data. Data collected through the Garmin devices were initially stored on a Health Insurance Portability and Accountability Act (HIPAA)–protected Amazon Web Services (AWS) server and then transmitted to a secure server at the Texas Advanced Computing Center, where they were processed and analyzed for this study.

### mHealth Platform

The mHealth platform enables long-term data collection from patients, including metrics related to functionality and fitness (physical activity patterns), autonomic function (heart rate variability), and sleep quality (latency and duration). The open-source app was built for smartphones using the Android operating system and has demonstrated high user compliance in prior research involving different clinical populations [11]. The prototype infrastructure leverages the HL7 Fast Healthcare Interoperability Resources (FHIR) standard, which provides representational state transfer (RESTful) access to clean, granular data. FHIR represents granular clinical concepts as a set of resources that can be accessed individually or in aggregate. The FHIR API supports queries at the individual patient and population level and enables the exchange of health records in highly readable XML and JSON formats. It also provides a large set of data models and vocabularies informed by real-world implementation experience. We used FHIR to integrate the trace data generated from patients’ use of the mHealth wearable devices with their EHR data, thus creating a longitudinal record of observational data for each patient enrolled in our study.

Figure 1 shows the overall architecture of the mHealth prototype platform developed and deployed to collect data from patients with CAD recruited at the academic medical center. Patients were monitored through web-connected or smartphone-connected devices, such as consumer fitness bands. The data were then piped to AWS nodes before being forwarded to the high-performance computing cluster at the research site and Amazon Simple Storage Service (S3). Researchers then analyzed the data using Amazon Elastic Compute Cloud (EC2). This infrastructure is highly scalable, always available, and extremely fault tolerant.

**Figure 1.**
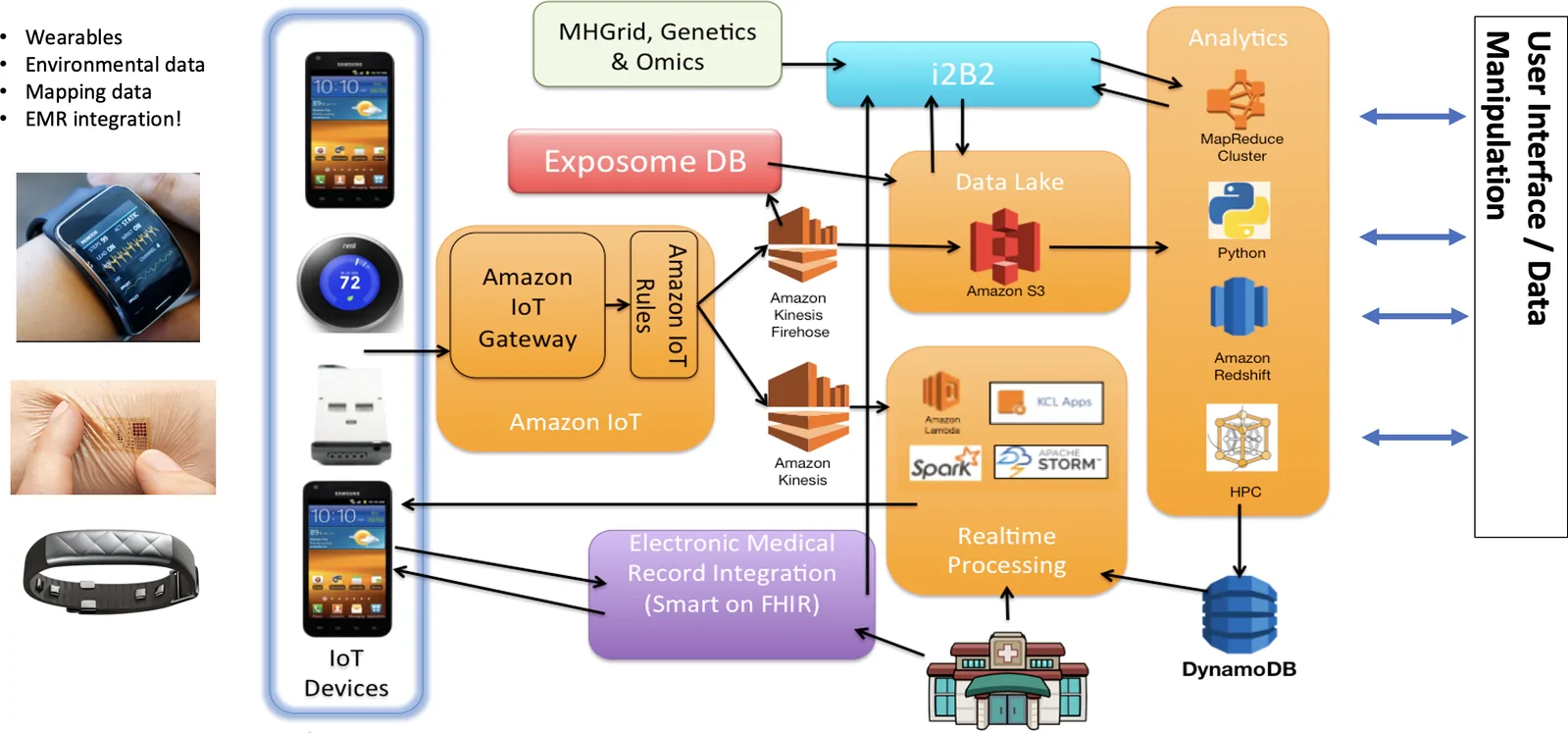
System design of the mobile health (mHealth) prototype platform developed to collect data from patients with coronary artery disease recruited into the study. The figure shows the technical components of the platform, as well as data flows among the patients’ wearable devices, backend cloud computing services, electronic health record integration, and data processing using a high-performance computing (HPC) cluster at the research site. EMR: electronic medical record; FHIR: Fast Healthcare Interoperability Resources; IoT: Internet of Things.

### Data Collection

We tracked patient health outcomes over a 2-year period, from June 1, 2019, to May 31, 2021. This period encompassed 18 months of data collected prior to the first wave of patient recruitment (which started in January 2021) and included over 5000 EHR encounters of patients’ interactions with the health care system. After restricting these records to clinical encounters, approximately 1500 health encounters remained across the pretreatment and posttreatment periods combined (here, treatment refers to the use of the mHealth prototype wearable device after recruitment into the study). These encounters included inpatient hospitalizations, outpatient visits, telehealth visits, and other encounters such as visits related to medication refills, procedures, and infusions. Of these, approximately 200 health care encounters occurred during participants’ use of the Garmin mHealth wearable devices.

The 18-month period between June 1, 2019, and December 31, 2020, served as the pretreatment period, allowing us to establish a baseline for evaluating patients’ health care encounters and utilization patterns prior to their recruitment into the study. Although the data primarily documented the timing and type of each encounter, a smaller subset also included patient measurements, such as height, weight, blood pressure, and pulse rate. The posttreatment period began in January 2021, when the first patients were enrolled in the study. Because recruitment occurred on a rolling basis, individual start dates were staggered over the following months. Hence, our treatment cohort included patients recruited into the study who used the mHealth prototype wearable device, with each patient’s posttreatment period defined relative to their enrollment date. Patient encounters with the health care system were documented in the EHR system of the cardiology clinic and matched to the wearable device data using a unique identifier.

### Ethical Considerations

Morehouse College served as the overarching institutional review board (IRB) (Protocol #862899‐14) for the participating academic institutions during the grant period. Patient recruitment and data collection were conducted at the University of Texas Southwestern Medical Center in accordance with the terms of the National Science Foundation grant (1636933) and prior IRB approval.

As patient recruitment and data collection were conducted primarily during and after the onset of the COVID-19 pandemic, the study received approval from the UT Southwestern Human Research Protection Program Reviewer (STU-2018‐0355) to conduct all study activities virtually. Patients were recruited through messages sent via the Epic MyChart app, followed by virtual visits with the study coordinator. We obtained verbal informed consent from all participants, and verbal authorization pertinent to HIPAA was also requested. The study materials were updated to reflect these changes.

A copy of the informed consent document used as part of the study protocol is provided in Multimedia Appendix 1. Verbal informed consent was obtained by the study coordinator at the time of patient recruitment. An addendum describing deviations from the study protocol owing to disruptions caused by the COVID-19 pandemic is also included in Multimedia Appendix 1.

All study data were anonymized or deidentified for this research. No identifiable study participant data have been reported in this manuscript. Data were identified by unique study IDs rather than by any personal identifier. The code linking participant IDs to identifying information was stored separately from the study data and was accessible only to trained research staff directly involved in the project. Identifiers were destroyed 12 months after study completion. Electronic data were stored in secure, password-protected computer files. All research staff were trained in safeguarding confidential data and protecting human participants, as required at the study site.

Participants did not receive monetary compensation for participating in the study. However, they were allowed to keep the Garmin wearable device provided at recruitment after the study ended.

## Results

### Statistical Analyses

Across the 20 participants initially enrolled in the study, we assembled 1904 patient-weeks of EHR data, including 263 patient-weeks of postenrollment data and 176 patient-weeks of wearable device data. Because participants were enrolled on a rolling basis from January to May 2021, the duration of wearable device observations varied across participants, with a median of approximately 8 patient-weeks per participant and 25th and 75th percentiles of 7 and 11 patient-weeks per participant, respectively. This variation reflected the staggered schedule of enrollment, which led to shorter follow-up periods for participants enrolled later. During the initial data quality assessment, we excluded 1 participant whose data did not meet the study’s reliability requirements. This exclusion resulted in a sample of 19 participants and 1803 patient-weeks of data, including 248 patient-weeks of postenrollment data. This sample was used for the descriptive time-trend analyses shown in Figures 2 and 3 and for subsequent pretreatment-posttreatment analyses.

**Figure 2.**
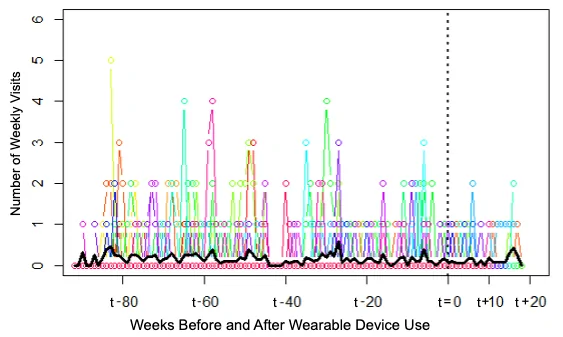
Time trends of weekly hospital visits by patients. Weekly hospital visit counts for 19 patients with coronary artery disease enrolled in a longitudinal observational study of mobile health (mHealth) Garmin wearable device use. Time is measured in weeks relative to the start of device use, with *t*=0 denoting the week in which a patient first began wearing the device. Negative values indicate weeks prior to device use, and positive values indicate weeks following device use. Colored dotted lines represent weekly hospital visit counts for individual patients, and the solid black line represents average weekly hospital visit counts across patients. The vertical dotted line identifies the transition from the preparticipation period to the postparticipation period.

**Figure 3.**
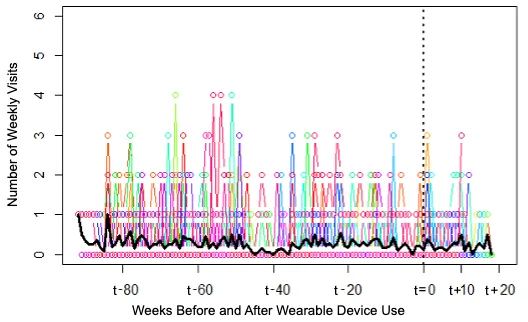
Time trends of weekly outpatient visits by patients. Weekly outpatient visit counts for the cohort of 19 patients with coronary artery disease enrolled in a longitudinal observational study of mobile health (mHealth) Garmin wearable device use. Visits are plotted relative to the start of wearable device use, with *t*=0 indicating the week of initial device adoption. Negative and positive time values correspond to preparticipation and postparticipation periods, respectively. Colored dotted lines denote weekly outpatient visit counts for individual patients, and the solid black line shows the average weekly outpatient visit counts across patients. The vertical dotted line identifies the transition from the preparticipation period to the postparticipation period.

To examine associations between physiological measures derived from mHealth device use and subsequent health care utilization, we matched the wearable data with EHR data by participant and patient-week. Patient-weeks with incomplete wearable data were excluded, reducing the 176 available patient-weeks to 131 complete, matched patient-weeks. Most exclusions resulted from missing sleep measures because participants removed their devices overnight despite being asked to wear them throughout the study period. These 131 patient-weeks constituted the sample used to summarize the weekly wearable measures in Table 1 and to estimate the regression models of physiological measures described below.

**Table 1.** Descriptive statistics of weekly patient physiological data^a^.

	Mean (SD)	Median	Maximum	Minimum
Patient-week wearable device data				
Active kilocalories burned	1610.10 (977.76)	1698	4342	60
Steps (thousands)	36.78 (19.78)	37.52	72.61	3.38
Active time (hours)	14.58 (8.37)	14.59	34.18	1.30
Mean heart rate (bpm^b^)	74.36 (5.67)	74.72	85.06	60.25
Median heart rate (bpm)	72.53 (5.80)	73	84	59
SD of heart rate (bpm)	11.19 (1.91)	11.34	16.14	7.03
Maximum heart rate (bpm)	133.56 (18.47)	133	193	102
Minimum heart rate (bpm)	51.33 (5.31)	52	62	38
Mean stress (Garmin scale, 1‐100)	14.81 (4.28)	14.52	26.38	6.00
Median stress (Garmin scale, 1‐100)	7.153 (5.57)	7	21	0
SD of stress	18.83 (3.74)	18.65	28.48	10.30
Maximum stress (Garmin scale, 1‐100)	91.24 (5.80)	93	99	76
Minimum stress (Garmin scale, 1‐100)	0 (0)	0	0	0
Total sleep time (hours)	41.19 (11.99)	45.45	61.35	3.98
Light sleep time (hours)	27.86 (8.10)	29.65	46.83	3.82
Deep sleep time (hours)	4.57 (3.48)	4.05	13.98	0
Pretreatment baseline (patient-level)				
BMI (kg/m²)	29.47 (6.34)	26.96	42.05	20.70
Systolic BP^c^ (mmHg)	122.62 (9.90)	123.56	144.33	102.44
Diastolic BP (mmHg)	76.29 (6.81)	73.95	93.89	67.33
Heart rate (bpm)	74.64 (9.03)	73.33	91.14	57.86

a This table reports descriptive statistics for physiological measures collected from 19 patients with coronary artery disease during the posttreatment period, based on their use of a mobile health (mHealth) wearable device. The physiological measures include active calories burned, number of steps, active time, heart rate, stress, and sleep time. Posttreatment data were tracked and collected between January and May 2021 and included 131 patient-weeks of observational data recorded via the mHealth device. The table reports the mean, median, SD, maximum, and minimum values for each attribute. It also presents baseline vitals, including BMI, blood pressure, and heart rate, recorded at the time of patient enrollment.

b bpm: beats per minute.

c BP: blood pressure.

Table 1 presents descriptive statistics measured at 2 levels. The first panel summarizes data collected from weekly wearable device use using the sample of 131 matched patient-weeks. The second panel reports participants’ baseline vital signs (body mass index, blood pressure, and heart rate) for the 19 participants. Although the original study protocol reported in Multimedia Appendix 1 specified monthly collection of vital signs, disruptions caused by the COVID-19 pandemic prevented these measurements from being collected regularly. These measures were too sparsely observed to be included as time-varying covariates. Therefore, each participant’s baseline vitals at the time of enrollment were used for the analyses, as reported in the bottom panel of Table 1.

### Care Utilization

Next, we evaluated time-variant patterns in the incidence of health encounters based on the number of weeks before and after patient participation. Figure 2 displays the average weekly hospital visit counts for the 19 participants. The horizontal axis shows time in weeks before and after study participation, with *t*=0 representing the week in which a patient first began using the wearable device. Because patient enrollment occurred on a rolling basis, *t*=0 was defined relative to each patient’s individual start date rather than a shared calendar week, aligning all patients on a common study-time axis despite staggered enrollment. For example, *t*–20 and *t*+10 represent timelines 20 weeks before and 10 weeks after a patient’s first use of the wearable device, respectively. Similarly, Figure 3 shows the time plots of patients’ weekly outpatient visits. Dotted lines in different colors represent weekly hospital visit counts for each patient, whereas the solid black line represents the average number of weekly hospital visits in Figure 2 and outpatient visits in Figure 3.

The top panel in Table 2 summarizes the mean weekly number of patient visits for each encounter type in the pretreatment and posttreatment periods. We compared differences in health care utilization across the four types of health care encounters, before and after patient enrollment, based on patients’ use of the mHealth wearable device. The data presented in Table 2 pertains to 1803 patient-weeks, with 1555 and 248 patient-weeks corresponding to the pretreatment and posttreatment periods, respectively. This total is larger than the 131 patient-weeks reported in Table 1 as the analysis in Table 2 includes EHR data only (prior to matching these data with the mHealth wearable measurements).

**Table 2.** Pretreatment and posttreatment differences in patient health encounters^a^.

Period/model	Hospital visits	Outpatient visits	Other visits	Telehealth visits	All visits
Mean encounters by period					
Pretreatment period	0.163	0.262	0.085	0.331	0.841
Posttreatment period	0.103	0.213	0.137	0.373	0.825
Overall (n=1803)	0.155	0.255	0.092	0.337	0.839
Pretreatment and posttreatment comparisons using GLMMs					
Poisson					
Coefficient	−0.540	−0.325	0.429	0.011	−0.124
*z*	−2.67	−2.16	2.28	0.10	−1.64
*P* value	.01	.03	.02	.92	.10
Poisson+patient baseline vitals					
Coefficient	−0.539	−0.323	0.441	0.013	−0.123
*z*	−2.66	−2.15	2.34	0.11	−1.64
*P* value	.01	.03	.02	.91	.10
Poisson, COVID-19 period					
Coefficient	−0.341	−0.139	0.330	−0.074	−0.083
*z*	−1.60	−0.87	1.66	−0.62	−1.04
*P* value	.11	.38	.10	.54	.30

a This table reports associations between adoption of the mHealth wearable device and weekly health care utilization for 19 patients with coronary artery disease. The analysis is based only on patients’ electronic health record (EHR) data, including 1555 patient-weeks from the pretreatment period and 248 patient-weeks from the posttreatment period (for a total of 1803 patient-weeks). Visits are categorized as hospital, outpatient, telehealth, other (including refills, procedures, and infusions), or all visits combined. Each cell presents the posttreatment coefficient on the log-count scale from a generalized linear mixed model (GLMM) with a random patient intercept for repeated weekly observations; negative values indicate fewer posttreatment visits. Inference is based on 2-sided Wald *z* tests.

The bottom panel of Table 2 presents the estimated differences in weekly utilization for each encounter type between the pretreatment and posttreatment periods. We estimated GLMMs with a random patient intercept for repeated weekly observations. A negative coefficient indicates fewer visits in the posttreatment period, and its exponential, the rate ratio, shows the multiplicative change in expected weekly visits. The first row corresponds to the Poisson model, which regresses weekly visit counts on a posttreatment indicator and a random patient intercept. The random intercept accounts for the repeated weekly observations and unobserved, patient-specific heterogeneity. The second row (Poisson+patient baseline vitals) includes each patient’s baseline vitals, including BMI, systolic and diastolic blood pressure, and heart rate as covariates. The Poisson GLMMs were estimated in R, version 4.3.0 (R Foundation for Statistical Computing), using the *glmmTMB* package, version 1.1.11.

Statistically significant associations were noted for hospital, outpatient, and other visits at the .05 significance level. The posttreatment coefficient for hospital visits was −0.540 (*z*=−2.67, *P*=.01), corresponding to a 42% reduction in expected weekly hospital visits relative to the pretreatment baseline. Outpatient visits declined by a comparable proportion, with a coefficient of −0.325 (*z*=−2.16, *P*=.03), corresponding to a 28% reduction. In contrast, other visits, including those for prescription refills, procedures, and infusions, increased, with a coefficient of 0.429 (*z*=2.28, *P*=.02), corresponding to a 54% increase. Differences in telehealth visits and total visits were not statistically significant.

As shown in the second row of the bottom panel of Table 2, inclusion of baseline patient vitals collected at the time of patient enrollment yielded quantitatively similar results across all outcomes. Overall, these findings suggest that use of the mHealth prototype wearable device was associated with a reduction in inpatient and outpatient utilization, along with a concurrent increase in the utilization of other procedural health care services, including visits for preventative care.

Our estimates of changes in health care utilization were sensitive to the pretreatment, baseline period because it included several months of patient data collected before the onset of the COVID-19 pandemic. The COVID-19 pandemic represented an external shock, and its impact was difficult to disentangle from our pretreatment-posttreatment comparison analysis. This is because our pretreatment window (ie, June 2019-December 2020) spanned both the prepandemic and pandemic eras, whereas the posttreatment period (ie, January 2021 onward) fell almost entirely within the pandemic period. To address this concern, we conducted additional robustness analyses to reestimate the GLMMs while restricting the pretreatment period to the COVID-19 period only (ie, March 2020 onward). This ensured that both pretreatment and posttreatment analyses were based on a comparable pandemic-affected health care utilization environment.

The results for the COVID-19 period only are reported in the last row of Table 2. Statistical significance of the GLMM coefficients was attenuated for certain types of health care visits. For example, the significance of the estimated coefficients decreased from *P*=.01 to *P*=.11 for hospital visits, from *P*=.03 to *P*=.38 for outpatient visits, and from *P*=.02 to *P*=.10 for other visits. These results reflect the fact that the COVID-19 pandemic sharply suppressed hospital health care utilization before rebounding, which compressed the pretreatment-posttreatment differences when using only the pandemic-era data, thereby reducing statistical power rather than indicating the absence of an effect. Hence, we qualified the results reported in the bottom panel of Table 2 considering disruptions to care utilization patterns caused by the COVID-19 pandemic.

### Physiological Data From Wearable Devices

Next, we analyzed wearable sensor data collected through participants’ use of Garmin mHealth devices at the patient-week level. During the period for which data were available, coverage across patient-weeks was 95% for stress level and 75% for heart rate.

Five types of patient physiological data were considered in our analyses: number of steps taken, time spent physically active, heart rate, stress level, and sleep time. Sleep time included light sleep time, deep sleep time, remaining sleep time, and total sleep time.

Because we estimated conditional, fixed-effects Poisson models, the estimation sample for each lag specification was restricted to patients who contributed nonzero variation in the outcome within a particular lagged window. Patients with no within-patient variation in visit counts were excluded as they did not contribute any identifying information to the conditional likelihood. Consequently, the number of patient-weeks (N) and number of patients reported at the bottom of each regression table vary across columns. These values represent the effective estimation sample for the specific lag rather than the full study sample. The conditional, fixed-effects Poisson models were estimated in Stata/MP, version 17.0 (StataCorp), using the native *xtpoisson* command with patient-clustered robust standard errors.

### Hospital Visits

Table 3 presents the Poisson regression results for hospital visits as the dependent variable. Benjamini-Hochberg FDR corrections were performed in Stata/MP, version 17.0 using native commands. The 100 2-sided Wald *P* values were ranked from smallest to largest, and the corresponding Benjamini-Hochberg–adjusted q values were calculated using the standard step-up procedure. Associations with q<.05 were considered statistically significant after FDR correction.

**Table 3.** Poisson regression results for hospital visits^a^.

Independent variable	Week *t*	Week *t*+1	Week *t*+2	Week *t*+3	Week *t*+4
Steps (thousands)					
Coefficient	0.0233	−0.0708	0.0297	0.0269	−0.0839
*z*	0.33	−0.55	0.58	0.41	−0.91
*P* value	.74	.58	.56	.68	.36
Active time (hours)					
Coefficient	−0.1422	0.0227	−0.0087	−0.0963	0.2532
*z*	−0.73	0.07	−0.10	−0.43	0.89
*P* value	.47	.94	.92	.66	.37
Minimum heart rate (bpm^b^)					
Coefficient	−0.0720	−0.0460	0.0200	*0.2610*	−0.0650
*z*	−1.63	−0.50	0.34	*3.94*	−1.13
*P* value	.10	.62	.73	*<.001*	.26
*q*	—^c^	—	—	*.004*	—
Maximum stress (Garmin scale)					
Coefficient	−0.0110	0.0910	0.0730	0.0390	0.0420
*z*	−0.20	1.23	0.88	0.29	0.48
*P* value	.84	.22	.38	.77	.64
Sleep time (hours)					
Coefficient	0.0404	−0.0351	−0.0429	0.0431	0.0985
*z*	0.94	−1.15	−1.02	0.95	2.36
*P* value	.35	.25	.31	.34	.02
N^d^ (patient-weeks)	78	77	76	75	66
Patients^e^	8	8	8	8	7
Log-likelihood	−23.44	−21.43	−21.87	−18.59	−15.60

a This table summarizes results from conditional fixed-effects Poisson regression models to study the association between physiological activity measures and weekly hospital visits among patients with coronary artery disease. Hospital visit counts are measured weekly and regressed on contemporaneous and lagged physiological measures, including total steps, total active time, minimum heart rate, maximum stress, and total sleep time. Columns correspond to models using physiological measures from the same week *t* and 1-4 week leads (*t*+1 through *t*+4). All models include patient fixed effects to control for time-invariant patient characteristics and use robust standard errors clustered at the patient level. Coefficients represent log changes in the expected count of hospital visits. Statistical inference is based on Wald *z* tests from conditional fixed-effects Poisson models; exact 2-sided *P* values are reported in every cell. To account for potential false discoveries, Benjamini-Hochberg–adjusted q values are computed; associations surviving correction at q<.05 are shown in italics. Because patient fixed effects are eliminated through conditional likelihood, they are not estimated separately and do not consume residual degrees of freedom.

b bpm: beats per minute.

c Not applicable.

d N: conditional fixed-effects estimation sample (ie, patient-weeks contributing nonzero outcome variations within the lagged window).

e Patients: number of patients that exhibit within-patient identifying variation.

The regression results indicated a robust association between minimum heart rate and the number of hospital visits. Specifically, minimum heart rate in week *t* was positively associated with hospital visits in week *t*+3 (*z*=3.94, *P*<.001), and this association survived the Benjamini-Hochberg correction for multiple comparisons (q=.004), indicating it was unlikely to be a false discovery. Sleep time in week *t* was also positively associated with hospital visits in week *t*+4 at conventional significance levels (*z*=2.36, *P*=.02); however, this association did not survive correction for multiple testing and should therefore be interpreted with caution. Other variables, including number of steps taken, activity time, and maximum stress level, were not significantly associated with hospital visits at any time lead.

Although this study focused on the association between patient physiological data and care utilization, our results—particularly the robust finding for minimum heart rate—appear consistent with prior research reporting associations between higher heart rate and greater in-hospital mortality among patients [12,13], even after accounting for differences in the severity of their health conditions.

### Outpatient Visits

We estimated the model in Equation 2 using outpatient visits as the dependent variable. The Poisson regression results are presented in Table 4. Minimum heart rate in week *t* was positively associated with outpatient visits in week *t*+2 (*z*=3.36, *P*=.001), and this association survived the Benjamini-Hochberg correction for multiple comparisons (q=.026), indicating it was unlikely to be a false discovery. The positive coefficient suggests that elevated minimum heart rate was associated with higher outpatient utilization in the subsequent 2-week window. No other physiological measures, including steps taken, activity time, maximum stress, and sleep time, were significantly associated with outpatient visits robust to Benjamini-Hochberg correction, whether contemporaneously or at any of the 4 time lags examined.

**Table 4.** Poisson regression results for outpatient visits^a^.

Independent variable	Week *t*	Week *t*+1	Week *t*+2	Week *t*+3	Week *t*+4
Steps (thousands)					
Coefficient	0.0940	−0.0316	0.1088	−0.0203	−0.1066
*z*	1.37	−0.40	1.23	−0.28	−1.23
*P* value	.17	.69	.22	.78	.22
Active time (hours)					
Coefficient	−0.4097	0.0801	−0.3484	0.1188	0.3207
*z*	−1.86	0.35	−1.68	0.56	1.45
*P* value	.06	.72	.09	.58	.15
Minimum heart rate (bpm^b^)					
Coefficient	0.0960	−0.1270	*0.1370*	−0.0550	−0.0030
*z*	1.63	−1.52	*3.36*	−0.78	−0.04
*P* value	.10	.13	*.001*	.44	.96
*q*	—^c^	—	*.026*	—	—
Maximum stress (Garmin scale)					
Coefficient	0.0570	−0.0230	0.0400	−0.0250	−0.0970
*z*	0.87	−0.48	0.45	−0.43	−1.07
*P* value	.38	.63	.65	.67	.28
Sleep time (hours)					
Coefficient	0.0009	−0.0026	0.0134	−0.0307	0.0051
*z*	0.04	−0.18	0.36	−1.60	0.11
*P* value	.97	.85	.72	.11	.91
N^d^ (patient-weeks)	85	91	87	92	89
Patients^e^	10	11	10	11	11
Log-likelihood	−39.92	−41.80	−37.27	−35.12	−33.23

a This table reports estimation results from conditional fixed-effects Poisson regression models examining the association between physiological activity measures and weekly outpatient visits among patients with coronary artery disease. Outpatient visit counts are measured weekly and regressed on contemporaneous and lagged physiological measures, including total steps taken, total active time, minimum heart rate, maximum stress, and total sleep time. Columns correspond to models using physiological measures from the same week *t* and from 1- to 4-week leads (*t+*1 through *t+*4). All models include patient fixed effects to control for time-invariant patient characteristics and use robust standard errors clustered at the patient level. Coefficients represent log changes in the expected count of outpatient visits. Statistical inference is based on Wald *z* tests from conditional fixed-effects Poisson models; exact 2-sided *P* values are reported in every cell. To account for potential false discoveries, Benjamini-Hochberg–adjusted q values are computed; associations surviving correction at q<.05 are shown in italics. Because patient fixed effects are eliminated through conditional likelihood, they are not estimated separately and do not consume residual degrees of freedom.

b bpm: beats per minute.

c Not applicable.

d N: conditional fixed-effects estimation sample (ie, patient-weeks contributing nonzero outcome variations within the lagged window).

e Patients: number of patients that exhibit within-patient identifying variation.

Our results regarding the association between minimum heart rate and frequency of hospital and outpatient visits are consistent with extant research. A recent study found that higher heart rate was associated with greater in-hospital mortality among patients in medical and surgery wards [12]. Among older patients hospitalized with heart failure, higher heart rate at discharge was associated with increased risks of death and rehospitalization, especially within the first 30 days after discharge [13]. Higher minimum heart rate may reflect underlying patient health risks, which could explain future hospital and outpatient visit utilization.

### Telehealth Visits

We estimated the model using telehealth visits as the dependent variable. The Poisson regression results are presented in Table 5. Several coefficients were statistically significant at conventional levels: maximum stress in week *t* (*z*=–2.64, *P*=.008), minimum heart rate in week *t*+2 (*z*=2.47, *P*=.01), and active time in *t*+4 (*z*=2.34, *P*=.02). However, none survived the Benjamini-Hochberg FDR correction for multiple comparisons and were thus not interpreted as robust associations. Steps taken and sleep time were not significantly associated with telehealth visits. Given the lack of FDR-robust findings in this specification owing to the low statistical power of the sample, substantive conclusions could not be drawn regarding the relationship between physiological activity measures and telehealth utilization.

**Table 5.** Poisson regression results for telehealth visits^a^.

Independent variable	Week *t*	Week *t*+1	Week *t*+2	Week *t*+3	Week *t*+4
Steps (thousands)					
Coefficient	−0.0168	0.0537	0.0728	0.0113	−0.0923
*z*	−0.34	1.04	1.35	0.21	−1.72
*P* value	.73	.30	.18	.83	.09
Active time (hours)					
Coefficient	−0.0825	−0.1805	−0.2007	0.1385	0.3709
*z*	−0.40	−1.16	−1.27	0.82	2.34
*P* value	.69	.25	.20	.41	.02
Minimum heart rate (bpm^b^)					
Coefficient	0.0380	−0.1060	0.1610	0.0630	−0.1040
*z*	0.62	−1.44	2.47	0.85	−1.39
*P* value	.54	.15	.01	.40	.16
Maximum stress (Garmin scale)					
Coefficient	−0.1110	0.1020	0.0290	0.1520	−0.0070
*z*	−2.64	1.67	0.51	1.86	−0.13
*P* value	.008	.09	.61	.06	.90
Sleep time (hours)					
Coefficient	0.0682	−0.0434	0.0089	−0.0501	−0.0281
*z*	1.59	−1.53	0.36	−1.01	−1.02
*P* value	.11	.13	.72	.31	.31
N^c^ (patient-weeks)	97	80	79	84	83
Patients^d^	11	10	10	10	10
Log-likelihood	−39.09	−36.51	−40.30	−39.16	−38.77

a This table summarizes results from conditional fixed-effects Poisson regression models examining the association between physiological activity measures and weekly telehealth visits among patients diagnosed with coronary artery disease. Telehealth visit counts are measured weekly and regressed on contemporaneous and lagged physiological measures, including total steps taken, total active time, minimum heart rate, maximum stress, and total sleep time. Columns correspond to models using physiological measures from the same week *t* and from 1 to 4 week leads, *t+*1 through *t+*4. All models include patient fixed effects to control for time-invariant patient characteristics and use robust standard errors clustered at the patient level. Coefficients represent log changes in the expected count of telehealth visits. Statistical inference is based on Wald *z* tests from conditional fixed-effects Poisson models; exact 2-sided *P* values are reported in every cell. To account for potential false discoveries, Benjamini-Hochberg–adjusted q values are computed; no associations survived FDR correction at q<.05. Because patient fixed effects are eliminated through conditional likelihood, they are not estimated separately and do not consume residual degrees of freedom.

b bpm: beats per minute.

c N: conditional fixed-effects estimation sample (ie, patient-weeks contributing nonzero outcome variations within the lagged window).

d Patients: number of patients that exhibit within-patient identifying variation.

### Other Visits

Lastly, we estimated the model using other visits as the dependent variable. The Poisson regression results are presented in Table 6. These visits were procedural in nature and included medication refill requests and medical procedures, such as infusions. A strong, FDR-robust positive association was noted between active time and other visits 4 weeks later, in week *t*+4 (*z*=6.54, *P*<.001, q<.001), indicating that each additional hour of weekly active time was associated with a 34% increase in expected procedural and refill-related visits. This magnitude was likely inflated by the small identifying sample and should thus not be interpreted as a clinically meaningful effect. Several other coefficients exhibited conventional significance: minimum heart rate in week *t*+1 (*P*=.007), maximum stress in weeks *t*+1 and *t*+2 (*P*=.02 and *P*=.02, respectively), and sleep time in week *t*+1 (*P*=.006). However, none survived Benjamini-Hochberg correction and were thus not considered robust associations.

**Table 6.** Poisson regression results for other visits^a^.

Independent variable	Week *t*	Week *t*+1	Week *t*+2	Week *t*+3	Week *t*+4
Steps (thousands)					
Coefficient	−0.0698	0.0376	0.0182	−0.0474	−0.0586
*z*	−0.33	0.35	0.38	−0.38	−1.37
*P* value	.74	.73	.70	.70	.17
Active time (hours)					
Coefficient	0.2495	0.0779	−0.1040	0.2795	*0.2957*
*z*	0.36	0.23	−0.41	0.74	*6.54*
*P* value	.72	.81	.68	.46	*<.001*
*q*	—^b^	—	—	—	*<.001*
Minimum heart rate (bpm^c^)					
Coefficient	0.1720	−0.2160	−0.1140	−0.0330	0.0120
*z*	1.11	−2.71	−0.96	−0.36	0.21
*P* value	.27	.007	.34	.72	.83
Maximum stress (Garmin scale)					
Coefficient	−0.0330	0.2410	−0.1370	0.0530	0.0460
*z*	−0.37	2.42	−2.41	0.44	0.45
*P* value	.71	.02	.02	.66	.66
Sleep time (hours)					
Coefficient	0.1074	0.1672	0.0158	0.0125	−0.0771
*z*	0.88	2.74	0.36	0.28	−1.65
*P* value	.38	.006	.72	.78	.10
N^d^ (patient-weeks)	58	57	66	55	64
Patients^e^	6	6	7	6	7
Log-likelihood	−14.03	−10.98	−18.27	−18.03	−19.56

a This table summarizes results from conditional fixed-effects Poisson regression models examining associations between physiological activity measures and weekly counts of other visits among patients with coronary artery disease. Other visits primarily include medication refill requests and medical procedures such as infusions. Other visit counts are measured weekly and regressed on contemporaneous and lagged physiological measures, including total steps, total active time, minimum heart rate, maximum stress, and total sleep time. Columns correspond to models using physiological measures from the same week *t* and from 1- to 4-week leads (*t+*1 through *t+*4). All models include patient fixed effects to control for time-invariant patient characteristics and use robust standard errors clustered at the patient level. Coefficients represent log changes in the expected count of other visits. Statistical inference is based on Wald *z* tests from conditional fixed-effects Poisson models; exact 2-sided *P* values are reported in every cell. To account for potential false discoveries, Benjamini-Hochberg–adjusted q values are computed; associations surviving correction at q<.05 are shown in *italics*. Because patient fixed effects are eliminated through conditional likelihood, they are not estimated separately and do not consume residual degrees of freedom.

b Not applicable.

c bpm: beats per minute.

d N: conditional fixed-effects estimation sample (ie, patient-weeks contributing nonzero outcome variations within the lagged window).

e Patients: number of patients that exhibit within-patient identifying variation.

### Summary

The statistical analysis of health care utilization trends using explanatory regression models indicates that patients’ physiological data are associated with their health care utilization, usually with time lags, although the strength of evidence varies across outcome types. The most robust findings across all four outcomes are the positive association between minimum heart rate and hospital visits after 3 weeks (Table 3) and the positive association between minimum heart rate and outpatient visits after 2 weeks (Table 4), both of which survive FDR correction. Similarly, we find a robust positive association between active time and other visits after 4 weeks (Table 6). In contrast, none of the associations identified for telehealth visits (Table 5) survive correction. Moreover, several associations across outpatient and other visit models that meet conventional significance criteria are not FDR-robust owing to the small sample size and should thus be interpreted with caution.

Overall, our research suggests that physiological data generated “in the wild” using mHealth wearable devices can help explain trends in future health care utilization, including the frequency of inpatient, outpatient, and other procedural encounters [12,14]. Nevertheless, the statistical inferences are based on a small sample, and the observed relationships should be treated as associational rather than causal.

## Discussion

### Principal Findings

Our study demonstrates the feasibility of a novel mHealth prototype that integrates physiological data collected through wearable devices with care utilization data collected from the EHR system of a large academic medical center. Specifically, this pilot study highlights the feasibility of a novel mHealth prototype in analyzing health care utilization by collecting and evaluating relevant measures of care utilization among patients with CAD.

Our research also demonstrates associations between patients’ physiological data and future health outcomes, measured based on care utilization across different health care delivery settings. Specifically, this pilot study evaluates the relationship between mHealth use and patients’ utilization of health care resources, such as inpatient, outpatient, telehealth, and other visits. The presented insights may support the triple aim of health care reform, namely, better individual and population health and lower costs, through the design of effective mHealth interventions to improve patient care coordination and health outcomes [15].

Our longitudinal observational study is based on a small panel of 19 patients with a history of CAD recruited from the cardiology clinic of a major academic medical center. The participants provided consent, met the study eligibility criteria, and fulfilled the study requirements. Data were collected through participants’ use of an mHealth prototype wearable device, which recorded various types of physiological data, such as activity time, sleep behavior, stress, heart rate, and steps taken, over a period of 5 months. Our findings suggest that physiological data from mHealth wearable devices can be used to study associations with the frequency of future care utilization in outpatient and inpatient settings.

Within this small cohort of patients with CAD, the frequency of future hospital and outpatient visits during the study period was significantly lower than that during the prior 18-month period. Other visits, including those for medication refills, infusions, and medical procedures, were significantly higher during the study period. Although the relationship between patients’ use of the wearable device and frequency of health encounters is associational, the simultaneous drop in hospitalizations and increase in other visits suggests an overall change in patients’ awareness of their health conditions. The study participants are more likely to use wearable devices to monitor their health conditions which, in turn, is associated with a drop in the frequency of hospital visits but greater utilization of other types of health care services, such as procedures and prescription refills. The latter finding suggests greater utilization of preventative care, as medication refills and procedures underscore patients’ propensity to adhere to their treatment regimen and medication schedules by undergoing regular tests and medical checkups.

### Comparison With Prior Work

Our results provide preliminary evidence of significant associations between patient physiological measures and incidence of future health encounters [16]. Among the physiological measures collected from the mHealth wearable device, minimum heart rate and activity time emerge as the only explanatory variables with associations that survive FDR correction for multiple comparisons.

These associations point to a broader pattern of patient engagement with health care providers that spans multiple channels, including hospital and outpatient visits as well as procedural and refill-related encounters. Specifically, minimum heart rate is positively associated with subsequent hospital and outpatient visits, whereas activity time is positively associated with other visits (procedural and refill related). These findings suggest that different physiological signals may be informative for different types of care seeking behavior by patients.

### Limitations

This study should be interpreted in the context of practical constraints common to early stage mHealth research. First, patient recruitment was limited by the clinical setting, geographic location, and technology requirements for wearable device deployment, resulting in a focused cohort drawn from a single cardiology clinic of an academic medical center.

Second, the number of patient-weeks with usable physiological data was limited by attrition and missing data, further emphasizing the exploratory scope of our analysis. Given the small sample size, this pilot study should be treated as an exploratory analysis of the association between wearable physiological signals and health care utilization.

Third, the study period overlapped with the COVID-19 pandemic, during which health care delivery and utilization patterns changed substantially. Increased reliance on telehealth, deferred inpatient care, and broader shifts in patient behavior during this period may have influenced the observed utilization trends alongside wearable device use and should be validated with additional data collected during the post-pandemic period. Notably, these conditions reflect the real-world environment in which mHealth wearable technologies are increasingly being implemented and evaluated.

Fourth, the study may be subject to selection bias as the users who agreed to participate may have been more engaged in managing their health than those who declined. A technology bias may also have been introduced as data were collected only from users of Android smartphones.

Lastly, disruptions caused by the COVID-19 pandemic resulted in deviations from the study protocol. Specifically, certain types of data, such as participants’ food choices and text messages on the smartphone app, could not be tracked or collected, and some patients could not be observed for the entire 3-month duration, as initially planned. Inclusion of such data in future studies would help improve the overall robustness of our preliminary findings.

### Future Directions

Future studies should expand the scope of this study to increase the sample size and length of the study period to improve the overall robustness of our preliminary results. Future work can be aimed at examining the intensity and frequency of wearable device use as potential predictors of future health care utilization. Research can also be conducted on the determinants of mHealth use, as well as the potential role of patient demographic characteristics and social determinants of health in moderating the impact of physiological data on health care outcomes. Finally, identification of a similar group of patients who do not use mHealth wearable devices could help assess the relationship between patients’ physiological data (collected through in-person encounters) and future care utilization patterns.

### Conclusions

The pilot mHealth prototype for assessing patient engagement and care coordination is intended to provide a scalable platform and technological infrastructure that can support efforts to improve patient engagement with health care providers. Results derived using wearable device data collected “in the wild” from the mHealth prototype platform highlight the importance of patient physiological data in examining associations with future health outcomes based on the frequency of different types of health care encounters [16]. Specifically, we find that physiological data such as active time and minimum heart rate are associated with future hospital and outpatient visits. Our study underscores the importance of processing and monitoring trends within wearable device data and integrating these results to develop actionable alerts and clinical intelligence for remote patient monitoring [17].

## Supplementary material

10.2196/81922 Multimedia Appendix 1 Study protocol.

## Abbreviations

AWS: Amazon Web Services
CAD: coronary artery disease
EC2: Elastic Compute Cloud
EHR: electronic health record
FDR: false discovery rate
FHIR: Fast Healthcare Interoperability Resources
GLMM: generalized linear mixed model
HIPAA: Health Insurance Portability and Accountability Act
IRB: institutional review board
mHealth: mobile health
RESTful: representational state transfer
S3: Simple Storage Service

## References

[1] Bao C, Bardhan IR, Singh H, Meyer BA, Kirksey K (2020). Patient-provider engagement and its impact on health outcomes: a longitudinal study of patient portal use. MIS Q.

[2] Yu S, Chai Y, Chen H, Sherman SJ, Brown RA (2022). Wearable sensor-based chronic condition severity assessment: an adversarial attention-based deep multisource multitask learning approach. MIS Q.

[3] Leng L, Arévalo Avalos MR, Aguilera A, Lyles CR (2025). The impact of an adaptive mHealth intervention on improving patient-provider health care communication: secondary analysis of the DIAMANTE trial. JMIR Mhealth Uhealth.

[4] Kim DS, Schuetz N, Johnson A (2025). Unlocking insights: clinical associations from the largest 6-minute walk test collection via the my Heart Counts Cardiovascular Health Study, a fully digital smartphone platform. Prog Cardiovasc Dis.

[5] Cakmak AS, Lanier HJ, Reinertsen E (2019). Abstract 15444: passive smartphone actigraphy data predicts heart failure decompensation. Circulation.

[6] Ghose A, Guo X, Li B, Dang Y (2022). Empowering patients using smart mobile health platforms: evidence from a randomized field experiment. MIS Q.

[7] Nambiar R, Bhardwaj R, Sethi A, Vargheese R A look at challenges and opportunities of big data analytics in healthcare.

[8] Sohn A, Speier W, Lan E (2019). Assessment of heart failure patients’ interest in mobile health apps for self-care: survey study. JMIR Cardio.

[9] Bente BE, Wentzel J, Schepers C (2023). Implementation and user evaluation of an eHealth technology platform supporting patients with cardiovascular disease in managing their health after a cardiac event: mixed methods study. JMIR Cardio.

[10] Yeung AWK, Kulnik ST, Parvanov ED (2022). Research on digital technology use in cardiology: bibliometric analysis. J Med Internet Res.

[11] Osipov M, Behzadi Y, Kane JM, Petrides G, Clifford GD (2015). Objective identification and analysis of physiological and behavioral signs of schizophrenia. J Ment Health.

[12] Yamamoto M, Ohta Y, Sakuma M, Takeuchi J, Matsumoto C, Morimoto T (2019). Association between heart rate on admission and in-hospital mortality among general inpatients: Insights from Japan Adverse Drug Events (JADE) study. Medicine (Baltimore).

[13] Laskey WK, Alomari I, Cox M (2015). Heart rate at hospital discharge in patients with heart failure is associated with mortality and rehospitalization. J Am Heart Assoc.

[14] Bianchini E, Alborghetti M, Galli S (2025). Minimal clinically important difference of average daily steps measured through a consumer smartwatch in people with mild-to-moderate Parkinson disease: cross-sectional study. JMIR Mhealth Uhealth.

[15] Bates DW, Saria S, Ohno-Machado L, Shah A, Escobar G (2014). Big data in health care: using analytics to identify and manage high-risk and high-cost patients. Health Aff.

[16] Mouttham A, Peyton L, Kuziemsky C (2011). Leveraging performance analytics to improve integration of care.

[17] Reyna MA, Josef CS, Jeter R (2020). Early prediction of sepsis from clinical data: the PhysioNet/Computing in Cardiology Challenge 2019. Crit Care Med.

