# Supplementary material for: Health Care Utilization Among Patients With Coronary Artery Disease Using mHealth Wearable Devices: Feasibility Observational Study
Source: JMIR Cardio. 2026 Nov 13;10:e81922. doi: 10.2196/81922 (PMC13762680; doi:10.2196/81922)
Supplement: Supplementary file 1 [file cardio-v10-e81922-s001.docx]

## Multimedia APPENDIX 1: Study Protocol

### Verbal Informed Consent

Study Coordinator: Thank you for meeting with me today. Before we begin, I’d like to tell you about the study and why you are being asked to take part.

Researchers at the Academic Medical Center are conducting a study to improve heart disease outcomes for adults. We hope to give you tools to improve your heart health. We are looking for changes in heart health-related activities, like physical activity, sleep, food choices and emotional health. We want to see if a smartphone app can improve heart disease outcomes.

You are being asked to take part in this study because you are at least 21 years of age, have heart disease, and use an Android phone.

The purpose of this research is to measure a new way to improve healthy behaviors in adults with heart diseases with a smartphone app. If you choose to take part, we will ask you to download and use a smartphone app. You will get a Garmin device (like a Fitbit) that measures heart rate and activity. We ask that you use the device and app for up to three months. You can keep the wearable device as a thank you for taking part in the study.

At the beginning, we will record your blood pressure, height, weight, waist size, fat/blood sugar levels, and physical activity levels (movement and distance). We will also track your stress and food choices. We will collect this data every month during the project and again 2 months after the project ends. During the study, the smartphone app will have access to your text messages. We will not save the text messages- we will record how often you say certain health-related words, and how many people you text. At the end of the study, we will ask you to do a survey to assess your opinion of the app.

Taking part in this study is voluntary. You may refuse to answer any question, take a break, or stop at any time. Deciding not to take part will not affect the health care you receive. However, answering the questions implies that you volunteer to participate.

Any time patient information is collected there is a possible risk for loss of privacy. Every effort will be made to keep your information private, though, this cannot be guaranteed. This information will only be shared with research staff involved in this study.

If you have any questions about the project please call the project coordinator at ____. For questions about your rights as a research participant, contact the Institutional Review Board (IRB) office at ____.

Before you agree to participate, please ensure that you understand the information given today. Make sure that your questions have been answered to your satisfaction, and that you have freely decided to join this research study.

**Addendum**: Due to the disruption caused by the COVID-19 pandemic, it was not possible to adhere to some aspects of the study protocol, described above. For example, patients’ food choices were not tracked and it was not possible to observe each patient for the entire 3-month duration after enrollment, as initially planned. Further, the smartphone app did not have access to the text messages of the patients during the study. Vital signs were not collected at the planned monthly intervals and were documented inconsistently during the study period. Therefore, the analyses used each participant’s baseline vital signs recorded at the time of enrollment. Finally, the survey to be conducted at the end of the study was not administered and is reported as a further deviation from the study protocol.
